# Cpk2, a Catalytic Subunit of Cyclic AMP-PKA, Regulates Growth and Pathogenesis in Rice Blast

**DOI:** 10.3389/fmicb.2017.02289

**Published:** 2017-11-21

**Authors:** Poonguzhali Selvaraj, Qing Shen, Fan Yang, Naweed I. Naqvi

**Affiliations:** ^1^Temasek Life Sciences Laboratory and Department of Biological Sciences, 1 Research Link, National University of Singapore, Singapore, Singapore; ^2^School of Biological Sciences, Nanyang Technological University, Singapore, Singapore

**Keywords:** *Magnaporthe*, rice blast, cyclic AMP, protein kinase A, catalytic subunit, localization

## Abstract

The cAMP-Protein Kinase A signaling, anchored on CpkA, is necessary for appressorium development and host penetration, but indispensable for infectious growth in *Magnaporthe oryzae*. In this study, we identified and characterized the gene encoding the second catalytic subunit, *CPK2*, whose expression was found to be lower compared to *CPKA* at various stages of pathogenic growth in *M. oryzae*. Deletion of *CPK*2 caused no alterations in vegetative growth, conidiation, appressorium formation, or pathogenicity. Surprisingly, the *cpkA*Δ*cpk2*Δ double deletion strain displayed significant reduction in growth rate and conidiation compared to the single deletion mutants. Interestingly, loss of *CPKA* and *CPK2* resulted in morphogenetic defects in germ tubes (with curled/wavy and serpentine growth pattern) on hydrophobic surfaces, and a complete failure to produce appressoria therein, thus suggesting an important role for *CPK2-*mediated cAMP-PKA in surface sensing and response pathway. *CPKA* promoter-driven expression of *CPK2* partially suppressed the defects in host penetration and pathogenicity in the *cpkA*Δ. Such ectopic *CPK2* expressing strain successfully penetrated the rice leaves, but was unable to produce proper secondary invasive hyphae, thus underscoring the importance of CpkA in growth and differentiation *in planta*. The Cpk2-GFP localized to the nuclei and cytoplasmic vesicles in conidia and germ tubes. The Cpk2-GFP colocalized with CpkA-mCherry on vesicles in the cytosol, but such overlap was not evident in the nuclei. Our studies indicate that CpkA and Cpk2 share overlapping functions, but also play distinct roles during pathogenesis-associated signaling and morphogenesis in the rice blast fungus.

## Introduction

The Protein kinase A (PKA) family of Ser/Thr kinases is highly conserved in eukaryotes, and serves important phosphorylation-dependent functions in signal transduction and development (Hanks and Hunter, 1995). The PKA holoenzyme is an inactive heterotetramer composed of two regulatory (R) and two catalytic (C) subunits and the cooperative binding of two cAMP molecules to the R subunit frees and activates the C subunits to phosphorylate hundreds of targets and regulate a vast swath of biochemical and metabolic processes.

The catalytic subunit of PKA (PKA-C) is a typical structure for protein kinases and PKA signaling plays a central role in vegetative growth, development, mating, stress response, and pathogenicity in various fungi (Lengeler et al., 2000; D’Souza and Heitman, 2001). Multiple PKA isoforms are expressed in mammalian cells and have tissue-specific roles indicative of functional diversity. Three *TPK* genes encoding PKA-C were identified in the budding yeast *Saccharomyces cerevisiae*, and subsequently shown to share redundant and distinct functions in viability and in pseudohyphal morphogenesis, respectively (Toda et al., 1987; Robertson and Fink, 1998; Pan and Heitman, 1999). In *Candida albicans*, the catalytic isoforms Tpk1p and Tpk2p share positive roles in cell growth, while they have distinct roles in hyphal morphogenesis, stress response and regulation of glycogen metabolism (Sonneborn et al., 2000; Bockmühl et al., 2001; Cloutier et al., 2003). Meanwhile, numerous known filamentous fungi are found to possess only two distantly related PKA-C isoforms with varied functions (Lee et al., 2003; Banno et al., 2005; Ni et al., 2005; Schumacher et al., 2008). In most plant pathogenic fungi, deletion of one PKA-C isoform resulted in profound effects in virulence. For instance, of the two PKA catalytic subunits, only Adr1 kinase activity is essential for the dimorphic transition and pathogenesis in *Ustilago maydis* (Dürrenberger et al., 1998). Similarly, only one PKA isoform plays a predominant role in other phytopathogens like *Colletotrichum trifolii, C. lagenarium, Botrytis cinerea* and *Setosphaeria turcica* that utilize appressoria to penetrate and infect the host; or in *Mycosphaerella graminicola* and *Verticillium dahlia*, that invade the host through stomata or other natural openings (Yang and Dickman, 1999; Takano et al., 2001; Yamauchi et al., 2004; Mehrabi and Kema, 2006; Schumacher et al., 2008; Tzima et al., 2010; Hao et al., 2015).

The Rice Blast pathosystem has been extensively analyzed at the molecular level, and serves as a model in the study of plant–fungus interactions (Liu et al., 2013), and in tackling global food security (Nalley et al., 2016). The cAMP/PKA signaling in *M. oryzae* plays an important role in surface sensing, appressorium morphogenesis, turgor generation, and in regulating plant infection (Li et al., 2012; Yan and Talbot, 2016). Different components of the G-protein signaling such as the Gα, MagA, MagB or MagC, two Gβ (Mgb1 and Mgb2), a Gγ subunit, and the Rgs1 (regulator of G-protein signaling 1) have been characterized (Liu and Dean, 1997; Fang and Dean, 2000; Nishimura et al., 2003; Dean et al., 2005; Liu et al., 2007; Ramanujam et al., 2012). Anchoring and trafficking of G-protein signaling components on late endosomes endows *M. oryzae* with the ability to specifically activate, integrate and achieve modularity and spatio-temporal control of signaling responses critical for pathogenesis (Ramanujam et al., 2013). Downstream of the G proteins, the adenylate cyclase Mac1 (that synthesizes cAMP), its suppressor Sum1, and the cAMP phosphodiesterases have been characterized too (Choi and Dean, 1997; Adachi and Hamer, 1998; Ramanujam and Naqvi, 2010; Zhang et al., 2011). Mutants disrupted in the catalytic subunit gene *CPKA* exhibit normal growth and conidiation, but show delayed appressorium formation and loss of pathogenicity, which results from the defects in appressorial function (Mitchell and Dean, 1995; Xu et al., 1997). We showed that loss of the regulatory subunit of PKA (*RPKA*) results in complete loss of pathogenicity; and a suppressor mutant that partially restores the pathogenicity in *rpk*AΔ represents a point mutation in the *CPKA* locus (Selvaraj et al., 2017). Recently, the second catalytic subunit of PKA, Cpk2, has been characterized in *M. oryzae* (Li Y. et al., 2017). These studies confirm a crucial role for cAMP/PKA signaling in the development and pathogenicity of *M. oryzae.*

In this study, we set out to investigate the role of *CPK2* through gene-deletion analysis and functional characterization. To gain further insights into the function of cAMP-PKA signaling in *M. oryzae* pathogenicity, we also created a *cpk*A*cpk*2 double deletion mutant and also analyzed the subcellular localization of Cpk2. We show that the Cpk2 activity is largely redundant with CpkA, and that both the catalytic subunits act in concert to regulate hyphal growth and play overlapping roles in conidiation and appressorium formation in *M. oryzae*. Importantly, these processes are dependent on Cpk2, since *CPK*2 deletion removes even the residual virulence associated with loss of *CPK*A. The expression of *CPK*2 under the *CPK*A promoter, or the swapping in of *CPK*2 coding region for *CPK*A, restored the pathogenicity in *M. oryzae cpkA* null mutant. Unlike CpkA, the Cpk2 subunit localized predominantly to the nucleus in rice blast. Taken together, this study underscores the importance of cyclic AMP PKA signaling in the pathogenic differentiation of *M. oryzae*.

## Experimental Procedures

### Strains, Growth Conditions, and Transformation

The *Magnaporthe oryzae* strain B157 (wild type - WT) obtained from the Directorate of Rice Research (Hyderabad, India) and its transformants/derived strains were routinely cultured on prune agar medium (PA) at 28°C for 7–10 days. Preparations of the media, assessment of growth, conidiation and appressorium formation and *Agrobacterium tumefaciens*-mediated transformation (AtMT) were carried out as routinely (Ramanujam and Naqvi, 2010; Deng et al., 2012). Requisite transformants were screened by Southern blot analysis and/or locus-specific PCR and in each case, two confirmed strains were selected for further observations.

### Nucleic Acid Manipulation and Sequence Analysis

The *CPK*2 orthologs were identified by searching the Genbank and fungal genome databases using the BLAST program (Altschul et al., 1997) and multiple sequence alignments were carried out with ClustalW (Thompson et al., 1994) and Boxshade^1^. Plasmid DNA extractions and genomic DNA extraction from the complete medium (CM) grown mycelium were carried out using standard kits; Geneaid High Speed Plasmid Mini kit and Yeast DNA purification kit (Epicenter Biotechnologies, United States) according to the protocols mentioned therein. The PCR primers used in this study are mentioned in Supplementary Table S1. Nucleotide sequencing was performed using the ABI Prism big dye terminator method (PE Applied Biosystems). Southern blot analysis was performed by using the Enhanced chemiluminescent labeling and detection kit (Amersham Biosciences, RPN2108). Standard procedures were adopted for DNA restriction, agarose gel transformation and hybridizations for Southern blot (Sambrook et al., 1989).

### Generation of *cpk*A and *cpk*2 Deletion Mutants, Overexpression Strains and GFP Fusion Constructs

To generate deletion mutants of *cpk*A and *cpk*2, gene replacement vectors encoding glufosinate ammonium resistance in pFGL97 or the hygromycin resistance in pFGL44 flanking the respective ORF were constructed using ligation PCR approach and then transformed to WT using AtMT. To get *cpk*AΔ*cpk*2Δ, the *cpk*2Δ construct was introduced into *cpk*AΔ strain. The *CPK2*-GFP in pFGL820 (encoding sulfonyl urea resistance gene cassette) was constructed by sequential cloning of the eGFP ORF, the last 1kb and the downstream fragment of CPK2 ORF to yield the final construct pCPK2-GFP-Trpc construct (Selvaraj et al., 2017). The GFP-CPK2 overexpression construct was created by fusing the Moh3 promoter with the cpk2 ORF, sequentially cloned in to pFGL1010G which encodes sulfonyl urea resistance with an ilV locus which facilitate ectopic single copy integration (Yang and Naqvi, 2014). To construct a cpk2 ORF overlapping cpkA vector, the ORF of cpk2 was fused with the promoter of cpkA and ligated to pFGL880 (encoding sulfonyl urea resistance gene cassette) which already contained the *CPKA* 3′UTR.

### Protein Isolation and Western Blot Analysis

Total proteins (∼30 μg) from mycelia collected from 2 days old CM cultures extracted were separated on a 10% SDSPAGE gel and transferred to PVDF membranes for western blot analysis as described (Bruno et al., 2004; Liu et al., 2011). TEY phosphorylation of MAPKs was detected with the PhophoPlus p44/42 MAPK antibody kit (Cell Signaling Technology, Beverly, MA, United States) according to the manufacturer’s instructions. A monoclonal anti-actin antibody (Abcam) was used to detect actin.

### Plant Cultivar, Growth and Blast Infection Assays

Rice cultivar CO39 and barley cultivar Express susceptible to *M. oryzae* strain B157 were used for blast infection assays. Rice was grown at 80% humidity at 28°C and Barley was grown at 60% humidity at 24°C (day) and 22°C (night) with 12 h:12 h day:night cycles in a growth chamber. For plant infection assays, freshly harvested conidia at a concentration of 1 × 10^6^ /ml in 0.2% gelatin were used. Plant inoculation, incubation and lesion examination were conducted as mentioned previously (Ramanujam and Naqvi, 2010; Bosch et al., 2012; Selvaraj et al., 2017). Rice leaf sheath infection assay was performed as described (Kankanala et al., 2007). Surface-sterilized rice seeds germinated and grown in direct contact with the fungal mycelial plugs were examined for black or browning lesions in the roots after 2 weeks to assess the root infection (Dufresne and Osbourn, 2001).

### Real Time qRT-PCR Analysis

Total RNA isolation from the mycelia or the frozen germlings at different time points and plant samples was carried out using RNeasy Plant Mini kit (QIAGEN, United States). The first strand cDNA synthesis and qRT-PCR were performed as mentioned previously (Patkar et al., 2012). All qRT-PCR reactions were conducted twice with three replications for each sample using the requisite primer sets for open reading frames for *cpk*A, *cpk*2 and β-tubulin (*TUB2*) mentioned (Supplementary Table S1). The abundance of the gene transcripts was calculated by the 2^-ΔΔ*C*_T_^ method with β-tubulin as the internal control.

### Assays for cAMP-Dependent Protein Kinase A (PKA) and Quantification of Intracellular cAMP

PKA assay was performed using a non-radioactive cAMP-dependent protein kinase assay system fluorescent using the PKA model substrate, Kemptide (Promega, Madison, WI, United States), following the manufacturer’s instructions and the sample preparation, determination of protein concentration were carried out were prepared as mentioned previously (Kang et al., 1999; Selvaraj et al., 2017). The samples for cAMP estimation were prepared as described previously (Liu et al., 2007; Ramanujam and Naqvi, 2010), and the assays were carried out using the cAMP Biotrak Immuno-assay system (Amersham Biosciences, Piscataway, NJ, United States) according to the manufacturer’s protocol.

### Microscopy, Image Analysis and Processing

Staining with DAPI (diamidino-2-phenylindole; Sigma–Aldrich, United States) was carried out essentially as described already (Patkar et al., 2010; Ramanujam and Naqvi, 2010). Bright field and epifluorescence microscopy was performed with an Olympus IX71 or BX51 microscope (Olympus, Tokyo, Japan) using a Plan APO 100X/1.45 or UPlan FLN 60X/1.25 objective and appropriate filter sets. Images were captured with Photometrics CoolSNAP HQ camera (Tucson, AZ, United States) and processed using MetaVue (Universal Imaging, Downingtown, PA, United States), and Adobe Photoshop 7.0.1 (Mountain View, CA, United States). Time-lapse or live cell fluorescence microscopy was performed using a an UltraView RS-3 spinning disk confocal system (PerkinElmer Inc., United States) using a 491 nm 100 mW and a 561 nm 50 mW laser illumination under the control of MetaMorph Premier Software (Ramanujam et al., 2013,BR52). Typically, z-stacks consisted of 0.5 μm-spaced planes for every time point. Image processing and preparation was performed using Fiji^2^, and Adobe Photoshop.

### Statistical Analyses

The one-way analysis of variance (ANOVA) tests were performed in order to assess whether the differences between the average responses of the treatments were significant. *P* values lower than 0.05 were considered to be significant. Statistical data was analyzed by the Student’s *t-*test to ascertain the significance of individual treatments and replicates, wherever applicable.

## Results

### Identification and Gene-Deletion Analysis of *CPK2* in *M. oryzae*

Analysis of the genome sequence of *M. oryzae*^3^ revealed an open reading frame (ORF) that encodes a catalytic subunit of PKA, *CPK2* (MGG_02832; contig 6–2325: coordinates 2566–1350), which was distinct from the *CPKA* locus. *CPK2* encodes a member of the Class II PKA subunits unique to filamentous fungi (Schumacher et al., 2008). MGG_02832 (GI: 2682385) showed the presence of three exons spanning a 1474 bp ORF, predicted to encode a 408-amino acid polypeptide (XP_003720907.1). The Cpk2 protein contains a typical serine/threonine kinase domain as well as a C-terminal AGC domain, which is representative of a large family of kinases and is conserved in numerous PKA catalytic subunits (Pearce et al., 2010). *M. oryzae* Cpk2 shows 40–53% amino acid identity to Class II PKAs in yeast (Toda et al., 1987), *A. fumigatus* (Liebmann et al., 2004), *B. cinerea* (Schumacher et al., 2008), and *U. maydis* (Dürrenberger et al., 1998). A detailed phylogenetic analysis of the class II PKAs, including the *M. oryzae* Cpk2, has been described previously (Schumacher et al., 2008). The CpkA and Cpk2 proteins of *M. oryzae* share approximately 48% sequence identity, with the highest degree of divergence within the N-terminal region. The highly conserved protein kinase domain of Cpk2 extends from 75 and 350 aa and the nucleotide binding site LGTGFARV (81–89 aa) differed by single aa from the conserved motif within CpkA, whereas the active catalytic domain RDLKPEN (203–215 aa) was identical (**Supplementary Figure S1**).

We carried out gene-deletion analyses to determine the relative contributions of each catalytic subunit of PKA, *CPK*A and *CPK*2, to the pathogenicity of *M. oryzae.* In addition, a strain deficient for both *CPK*A and *CPK*2 was generated illustrating that cAMP-PKA signaling is not essential for viability in *M. oryzae*. The transformants were confirmed through Southern blotting and locus specific PCR (**Supplementary Figure S2**), and in all cases at least two independent strains were characterized in detail.

### Cpk2 and CpkA Are Required for Vegetative Growth and Conidiation in *M. oryzae*

**FIGURE 1:**
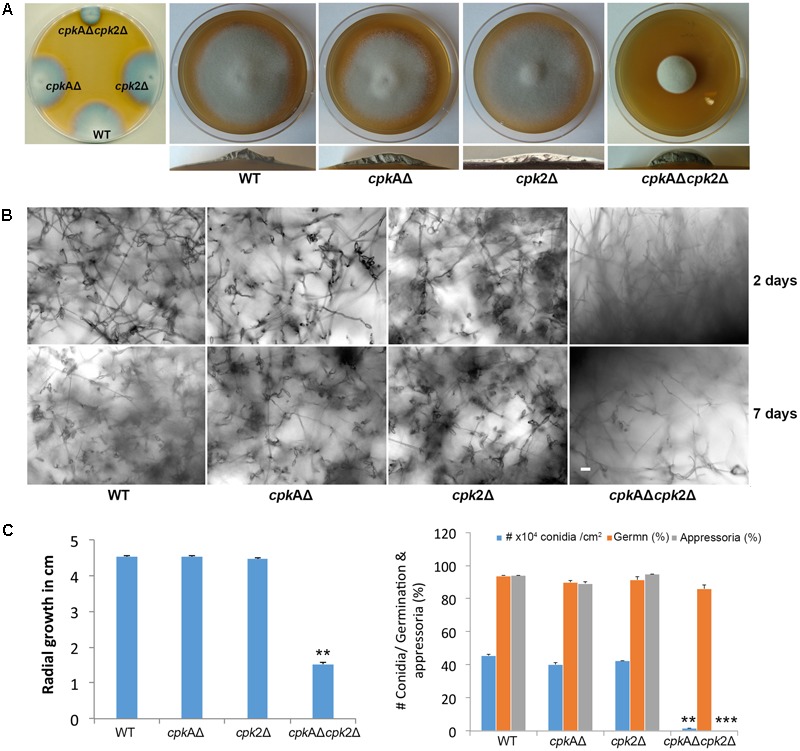
Cpk2-mediated cAMP-PKA signaling is necessary for proper vegetative and asexual development in *M. oryzae* **(A)** Radial and aerial hyphal growth of the wild type (WT) and the indicated PKA-C mutant strains. Mycelial plugs inoculated on PA medium was cultured in the dark at 28°C for 5 days. The left panel shows the comparative radial growth of individual CPK mutants *cpkA*Δ, *cpk2*Δ and *cpkA*Δ*cpk2*Δ with the WT B157. The radial and cross-sectional view for the aerial hyphal growth of WT and the mutants are shown (right). **(B)** Bright field micrographs showing the conidiation at 48 h and 7 days post photoinduction. Individual *cpk*AΔ and *cpk*2Δ produced conidia normally as the WT at 48 h, while *cpk*AΔ*cpk*2Δ showed very few conidia even at 7 dpi. Scale bar = 10 μm. **(C)** Bar graphs showing the difference in radial growth (left) and the quantification of conidiation and appressorium formation in WT and PKA-C mutants (right). Values represent mean ± SE of three independent replicates with approximately 200 conidia assessed per experiment; ^∗∗^*p* < 0.001, ^∗∗∗^*p* < 0.0001.

The colony morphology and the radial growth of the individual *cpk*AΔ and *cpk*2Δ strains were indistinguishable from the WT, while the *cpkA*Δ*cpk2*Δ showed reduced radial growth producing small colonies with fluffy aerial growth (**Figures 1A,C**). Although *CPKA* is dispensable for vegetative growth and conidiation, it regulates appressorium formation and function, with the *cpkA*Δ strain displaying long germ tubes and delayed appressorium formation (Mitchell and Dean, 1995; Xu et al., 1997). No apparent changes were observed in conidiation in *cpk*AΔ and *cpk*2Δ compared to the WT. Based on the *cpk*2Δ phenotypes, we inferred that Cpk2 functionality could be elucidated better in the context of the loss of CpkA activity. Accordingly, the deletion of *cpk2* in *cpk*AΔ, (*cpk*AΔ*cpk*2Δ), led to further delay and reduction in conidiation compared to the WT or the individual mutants. The WT, *cpk*AΔ or *cpk*2Δ strains produced conidia within 24 h of light exposure, whereas the *cpk*AΔ*cpk*2Δ mutant did not initiate conidia formation even after 48 hpi. At 7–10 days of exposure to light, the double mutant produced about 10-fold lesser (*p* < 0.01) conidia than the WT (**Figures 1B,C**). However, the conidia produced were three celled and with no apparent abnormalities in shape or size, thus implying that complete loss of PKA activity would adversely affect asexual development. We conclude that cAMP-PKA activity is essential for conidiation in *M. oryzae;* and that CpkA and Cpk2 possess overlapping roles in regulating such metabolic activation and initiation of asexual reproduction therein.

### *CPK2* Plays a Significant Role in Appressorium Formation in *M. oryzae*

**FIGURE 2:**
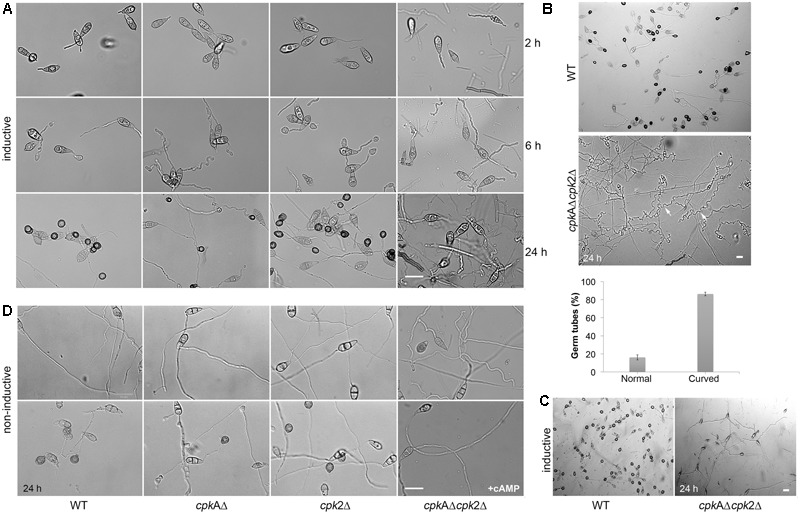
Cpk2 plays a crucial role in appressorium formation. Bright field micrographs of the appressoria formed by WT and CPK mutants on inductive – hydrophobic **(A)** and non-inductive - hydrophilic surfaces **(D)** at different time points. **(A)** The *cpk*AΔ*cpk*2Δ did not produce appressoria on inductive surface even at 24 hpi, while *cpk*AΔ was significantly delayed in appressorium formation. **(B)** The *cpkA*Δ*cpk2*Δ produced very long, wavy germ tubes without forming appressoria, and the germ tubes showed curled/zig-zag growth unlike the straight/linear growth mode in the WT. Different phenotypes of the germ tube formed by cpkAΔ*cpk*2Δ were quantified and represented as a bar graph. **(C)** Addition of exogenous cAMP (10 mM) to the conidia suppressed the wavy growth pattern, but failed to restore the appressoria formation in the *cpk*AΔ*cpk*2Δ. **(D)** Addition of exogenous cAMP (10 mM) to the conidia restored the appressorium formation in WT and *cpk*AΔ, *cpk*2Δ on non-inductive surface while *cpk*AΔ*cpk*2Δ was non-responsive to such exogenous cAMP. Scale bar = 10 μm.

The *cpk*AΔ showed a significant delay in appressoria formation on hydrophobic/inductive surfaces, and the appressoria produced were smaller with long germ tubes. The *cpk*2Δ produced normal appressoria indistinguishable from WT. Interestingly, the *cpk*AΔ*cpk*2Δ conidia germinated normally, but failed to elaborate appressoria on inductive surfaces even with excess cAMP. Germ tubes produced by *cpk*AΔ*cpk*2Δ conidia were very long, curled, showed periodic clockwise twists, and did not produce appressoria even at 32 hpi (**Figures 2A–C**). Unlike the WT, *cpk*AΔ or *cpk*2Δ that elaborate appressoria on non-inductive surfaces in response to exogenous cAMP, the *cpk*AΔ*cpk*2Δ was non-responsive to the cAMP stimulus and failed to elaborate appressoria (**Figure 2D**). Appressorium morphogenesis is tightly regulated by the cell cycle, with DNA replication and one round of mitosis being essential for the initiation of appressoria in *M. oryzae* (Veneault-Fourrey et al., 2006; Saunders et al., 2010; Li C. et al., 2017). We inferred that the defect in appressorium formation in the *cpk*AΔ*cpk*2Δ is likely due to the inability to sense and/or respond to cAMP in addition to defects in surface sensing and adhesion. The delayed appressorium formation in *cpkA*Δ, and the inability of the *cpk*AΔ*cpk*2Δ to initiate appressoria, further underscore the importance of *CPK2* in surface sensing and appressorium formation in *M. oryzae*.

To further clarify the role of *CPK*2, we added the cAMP-PKA inhibitor KT5720 to the conidia on coverslips and checked appressorium formation at 24 h. Addition of KT5720 delayed appressorium formation in the WT, but had no effect on appressorium morphology. KT5720 showed dose-dependent reduction in appressorium formation ability in *cpkA*Δ or *cpk2*Δ conidia. At 2 μM KT5720, appressorium formation decreased from 80 to 42% in the *cpk*AΔ (*p* = 0.004) compared to the solvent control, whereas the reduction was from 93 to 79% (*p* = 0.009) in *cpk2*Δ. At higher concentration (5 μM), the effect of KT5720 was highly variable and pleiotropic, with the resultant appressoria being malformed and/or underdeveloped; and the treated conidia did not show further reduction in the ability to form appressoria. The maximal inhibitor concentration used in our experiment was thus not sufficient to completely block the PKA activity in the WT strain. We conclude that albeit redundant, the second catalytic cAMP-PKA subunit, Cpk2, plays an important role in appressorium formation in the rice blast fungus.

### *CPKA* and *CPK2* Are Involved in Regulation of PKA Signaling and Intracellular cAMP Levels

**FIGURE 3:**
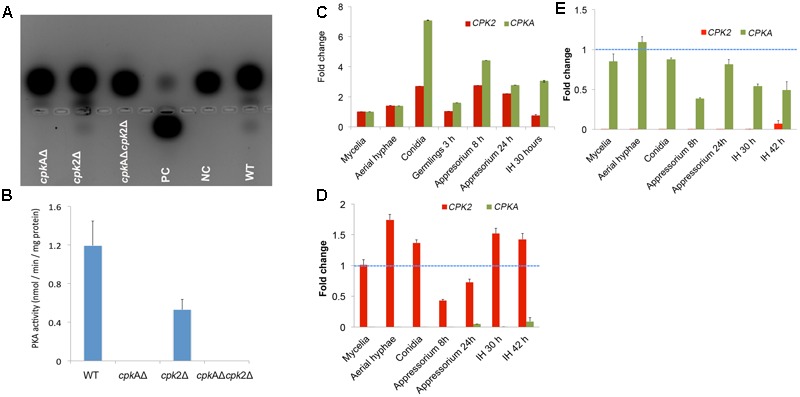
Catalytic activity and transcriptional regulation of the PKA-C subunits in *M. oryzae.* **(A)** PKA enzyme activity was monitored by gel electrophoresis showing the migration of phosphorylated substrate toward the anode. PC-positive control; NC-negative control. PKA activity was analyzed in the total protein extracts from the frozen mycelia of WT and the indicated CPK mutants. The results were consistent with repeated experiments. **(B)** Bar graph showing the PKA activity in WT and the listed PKA-C mutants. Each value represents a mean ± SE of three replications. Graphical representation of the fold change in the transcript/mRNA levels of *CPKA* and *CPK*2 in WT **(C)**, *cpk*AΔ **(D)** and *cpk*2Δ **(E)** at different stages of vegetative and pathogenic development. Reverse transcriptase RT-PCR was conducted on total RNA extracted from the mycelia and the infection structures mentioned. Fold change in gene expression in WT was calculated from the average of three independent measurements, with β-Tubulin of *M. oryzae* as internal control and normalized to unit mycelial biomass. For the mutants, for fold change the gene expression was normalized to WT levels (indicated by dotted blue line) at the respective phases with β-Tubulin of *M. oryzae* as internal control Error bars represent standard error. The experiment was repeated twice with three replicates each.

PKA activity was undetectable in the total protein extracts from the mycelia of *cpk*AΔ or *cpk*AΔ*cpk*2Δ, whereas the WT clearly showed the cAMP-dependent PKA activity (**Figure 3A**). PKA activity could still be detected in the mycelial extracts of *cpk*2Δ but was very low. The *cpk*2Δ produced only about 50% PKA activity *in vitro* compared to the WT *M. oryzae* (**Figure 3B**), indicating that there is no compensatory increase in CpkA activity in the absence of *CPK2.* However, the function(s) of Cpk2 cannot be ascertained solely on the basis of its enzyme activity; and the regulatory interactions and interdependency between the two isoforms could not be ruled out (Ni et al., 2005). Hence, we carried out qRT-PCR to determine the expression levels of *CPKA* and *CPK2* in order to check if deletion of *CPKA* affects the expression of *CPK2* or vice versa. In WT, albeit having a similar expression pattern, the level of *CPK*2 was comparatively lower than *CPKA* at all the time points tested (**Figure 3C**) indicating that the activity of CpkA alone contributes to the regular functions of cAMP-PKA in the *cpk*2Δ mutant. *CPKA* and *CPK*2 were expressed in mycelia and aerial hyphae at comparable levels, and hence the *cpk*AΔ*cpk*2Δ was highly impaired in radial and aerial growth. The expression of both isoforms increased in the appressoria at 8 h, which could be responsible for the indicated high levels of PKA activity during appressorium formation (Kang et al., 1999). Based on the higher levels of transcription of the PKA isoforms in conidia and appressoria, we infer their functional importance in conidia and appressoria formation and pathogenicity. In comparison to the WT, the expression level of *CPK2* in *cpk*AΔ did not show any change at any of the time points analyzed (**Figure 3D**). Similarly, the level of *CPKA* transcription was comparable in the *cpk*2Δ and the WT during the pathogenic phase (**Figure 3E**). The expression levels were comparatively higher in the aerial hyphae and conidia compared to the other phases of growth or pathogenesis. However, the overall increase was less than 2 fold, and therefore considered insignificant. Thus, we conclude that deletion of *CPKA* or *CPK2* had only a minor effect on the transcript levels of the other PKA isoform, thus ruling out the possibility of co-transcriptional regulation between *CPKA* and *CPK2* in *M. oryzae*.

**FIGURE 4:**
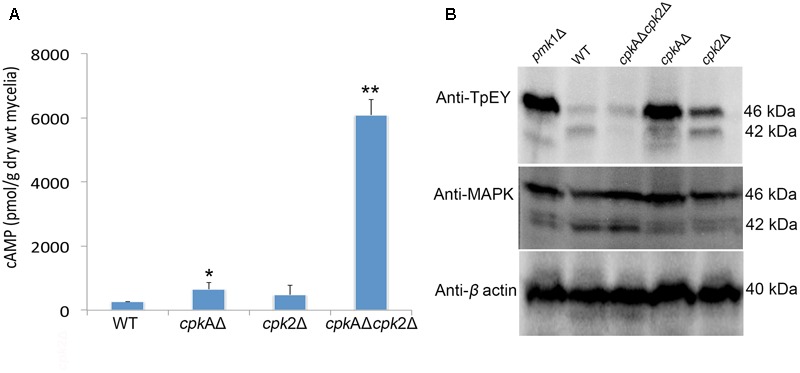
Protein kinase A (PKA) activity regulates the intracellular cAMP levels, and the Pmk1-MAPK signaling in *M. oryzae*. **(A)** Quantification of the intracellular cAMP levels in the mycelia of WT and the indicated mutant strains. Values from two biological replicates with two replications for each individual sample were analyzed and the mean value is presented; ^∗^*p* < 0.05. **(B)** Western blots showing the phosphorylation of Pmk1 (42 kDa) and Mps1 (46 kDa) MAPK in the WT and indicated PKA-C mutants. Total proteins were extracted from the mycelium of WT and mutants and analyzed with the indicated antibodies. The *pmk1*Δ served as a negative control and the experiment was repeated twice.

Compared to the WT, the PKA-C mutants showed increased intracellular accumulation of cAMP. The *cpk*AΔ showed higher cAMP levels than *cpk*2Δ, consistent with the predominant role for CpkA in overall PKA activity/function. However, *cpk2* deletion either in WT or *cpk*AΔ led to an increase in overall cAMP concentration indicating that Cpk2 also acts as a cAMP effector in *M. oryzae*. The cAMP increased to very high levels in *cpk*AΔ*cpk*2Δ (about 30-fold increase) indicating an additive effect of the loss of both catalytic subunits of cAMP-PKA (**Figure 4A**). We infer that cAMP-PKA activity limits the intrinsic cAMP to a threshold/moderate level to maintain normal cellular functions; or conversely, the activation of PKA dampens the intracellular cAMP pool. We conclude that Cpk2 acts in concert with CpkA to regulate the overall accumulation and dynamics of cAMP signaling in *M. oryzae*.

### Pmk1 MAPK Phosphorylation Is Affected in the cAMP-PKA Mutants

The non-responsiveness for cAMP in *cpk*AΔ*cpk*2Δ that resembles the *pmk*1Δ phenotype (Xu and Hamer, 1996; Kou et al., 2016) led us to assess whether the Pmk1 MAPK activation is compromised in the PKA-C mutants. Therein, we assayed the phosphorylation of Pmk1 with the anti-TpEY specific antibody that detects the phosphorylation of both Pmk1 and Mps1 MAPKs (Zhao et al., 2005). In WT, a band of 42 and 46 kDa indicating the phosphorylation of Pmk1 and Mps1 were observed, while in *pmk1*Δ only the Mps1 phosphorylation was evident (**Figure 4B**). A relatively weak phosphorylation of Pmk1 was evident in *cpk*AΔ, compared to the increased phosphorylation of Pmk1 in *cpk*2Δ; whereas such activation of Pmk1 MAPK was completely absent in the *cpk*AΔ*cpk*2Δ strain. The level of Mps1 phosphorylation increased in *cpk*AΔ or *cpk*2Δ compared to the WT, but similar to *pmk1*Δ indicating that Mps1 is likely hyperactive in response to/or to compensate for the cell wall defects associated with loss of Pmk1 (Zhao et al., 2005). However, the Mps1 phosphorylation remained stronger in *cpk*AΔ compared to *cpk*2Δ, likely as a consequence of the weak phosphorylation/activation of Pmk1 in *cpk*AΔ. The phosphorylation of Mps1 remained unaltered in cpkAΔ*cpk*2Δ when compared to WT *M. oryzae*. These results showed that signaling through Pmk1 may be impaired but not completely blocked in *cpk*AΔ or *cpk*2Δ, but an additive effect is observed in the double mutant wherein the total loss of Pmk1 activation is likely responsible for the observed defects in appressorium formation.

### cAMP-PKA Signaling and Pathogenesis of *M. oryzae*

**FIGURE 5:**
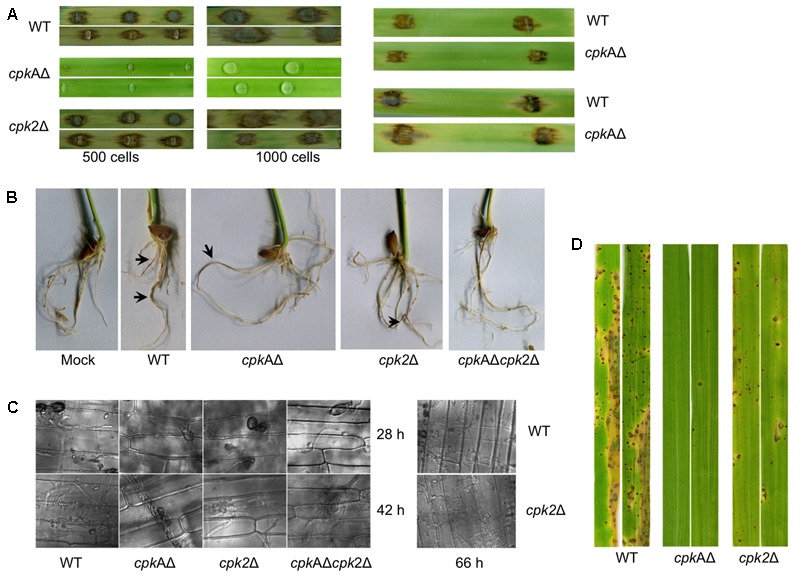
Protein kinase A signaling plays an essential role in the pathogenicity of *M. oryzae*. **(A)** Barley leaf infection assays with WT and the mutant strains. Conidia from WT or mutant strains were used to inoculate barley leaf explants and the blast disease symptoms/lesions were assessed 7 days post inoculation. Number of conidia used for each inoculation is indicated. For *cpkA*Δ, the abraded (wounded) leaves showed WT-like blast disease lesions (Right panel). **(B)** Rice root infection assays with WT and the PKA-C mutant strains. Surface sterilized rice seeds were allowed to germinate and grow on mycelial plugs of the WT or the mutant strains; and necrosis/lesions were recorded after an incubation of 15 days. Mock indicates PA plugs without the fungal cultures. Arrows indicate necrosis/lesions on the roots. **(C)** Bright field micrographs showing the invasive hyphal growth of WT and PKA-C mutants at the indicated time points when inoculated on rice leaf sheath. Scale bar = 10 μm. **(D)** Spray inoculation assays in rice to confirm the pathogenicity of WT and mutants. Conidia (1 × 10^5^/ml, 5 ml) from WT or PKA-C mutants were sprayed on 4-week-old seedlings of rice cultivar CO39. Blast disease symptoms were assessed at 10 dpi.

We tested the cAMP-PKA mutant strains for their ability to cause blast disease in barley and rice. The *cpk*AΔ and *cpk*AΔ*cpk*2Δ failed to elicit any visible blast symptoms on barley leaves, whereas the WT or *cpk*2Δ inoculation resulted in typical blast lesions on barley leaves. Wounding of rice or barley leaves with the micropipette tip helped the *cpk*AΔ to produce WT-like blast lesions in such abraded tissues (**Figure 5A**). While *cpk*AΔ was still able to elicit necrosis on rice roots comparable to the WT or *cpk*2Δ, the *cpk*AΔ*cpk*2Δ strain did not produce any visible disease symptoms or necrosis on rice roots (**Figure 5B**). Rice leaf sheath inoculations revealed that *cpk*2Δ was pathogenic, and was able to penetrate the host plants (28 hpi), and grow invasively into the neighboring cells similar to the WT (42–72 hpi). Consistent with previous results, the appressoria produced by *cpk*AΔ were impaired in penetration and could be observed on the surface of the rice leaf sheath at 28 and 42 hpi. The *cpk*AΔ*cpk*2Δ failed to produce appressoria on leaf sheath even at 42 hpi and hence was deemed completely non-pathogenic (**Figure 5C**). As observed with barley leaf assays, the spray inoculation of conidia from WT or *cpk*2Δ produced typical blast lesions on rice leaves, while *cpk*AΔ remained non-pathogenic (**Figure 5D**). Since *cpk*AΔ*cpk*2Δ produced very few conidia, we were unable to carry out spray inoculation assays for the double mutant strain.

### Swapping of the *CPKA* ORF with *CPK2*

**FIGURE 6:**
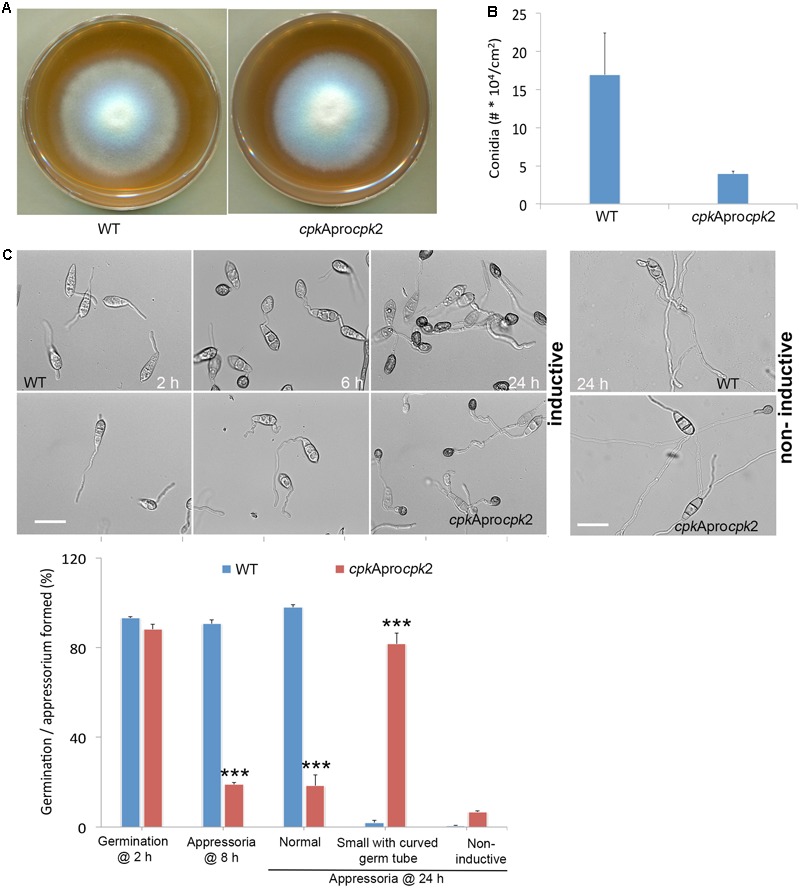
Cpk2 is able to partially compensate for the loss of CpkA. **(A)** Radial growth of CPKApromoter*CPK2 (cpk*Apro*cpk2)* compared with WT on prune-agar medium. The CPKApromoterCPK2 strain showed no defects in vegetative growth when compared to the WT *M. oryzae*. **(B)** Quantification of conidiation revealed that substituting *CPK2* open reading frame for CPKA results in reduced conidiation implicating a major role of *CPKA* in conidiation. Each bar represents mean ± SE of three independent replicates. **(C)** Appressorium formation on inductive (left) and non-inductive surfaces (right) and quantification of germination, appressorium formation by CPKApro*CPK2* compared with WT (lower panel). Values represent mean ± SE of three independent replicates using about 200 conidia per experiment. ^∗∗∗^*p* < 0.0001.

In order to check if Cpk2 could functionally complement CpkA, we precisely replaced the *CPKA* coding sequence with the *CPK2* ORF, thus creating a genetic background that consequently lacks *CPKA*, but expresses *CPK2* under the *CPKA* promoter/regulon. The native *CPK2* remained unperturbed in such swapped strain. The resultant *CPKA*_Promoter_*CPK*2 strain showed WT-like vegetative growth, but displayed up to tenfold reduction in conidiation, though the difference between the means was not significant (*p* = 0.07; **Figures 6A,B**). Furthermore, appressorium formation was delayed in the *CPKA*_Promoter_*CPK*2 strain, and the resultant appressoria formed after prolonged germ tube growth were smaller in size similar to the *cpk*AΔ (**Figure 6C**), again confirming that *CPKA* is required for proper appressorium formation. The PKA activity could not be detected in the total protein extracts from the mycelia of *CPKA*_Promoter_*CPK2* strain.

**FIGURE 7:**
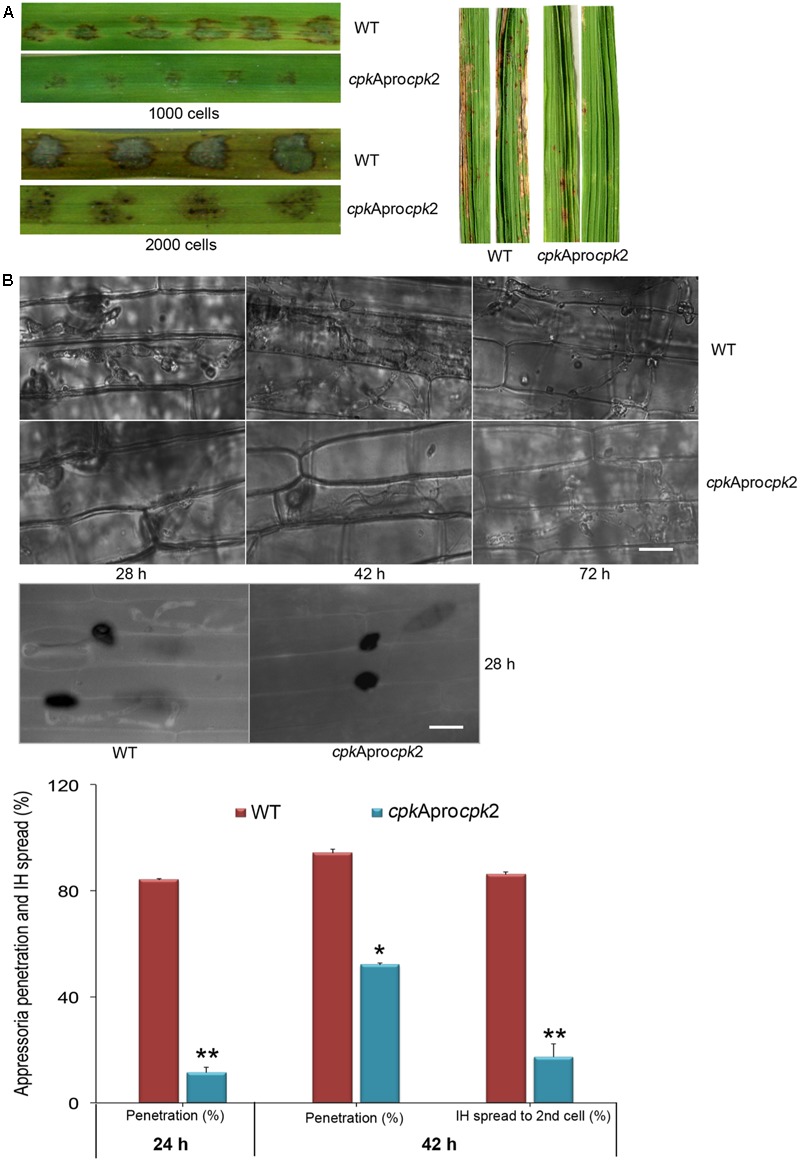
Substituting Cpk2 for CpkA partially suppresses the penetration and pathogenicity defects of *cpkA* deletion mutant. **(A)** Infection assays in rice with the conidia of CPKApromoter*CPK2* strain compared to the WT. Conidia from WT and CPKApromoter*CPK2* were used to spot inoculate rice leaves, or sprayed on to seedlings of rice cultivar CO39 (right). The number of conidia inoculated on rice leaves is indicated. Disease lesions were scored on 10 dpi for rice leaves and at 14 dpi for the spray inoculation assays. **(B)** Bright field micrographs showing the invasive hyphal growth of WT and CPKApromoter*CPK2* strain at the indicated time points (upper panel). Scale bar = 10 μm. The CPKApromoter*CPK2* showed impaired penetration and was defective in invasive hyphal growth. Micrographs showing host penetration by WT and *CPKA*_Promoter_*CPK2* at 28h stained with aniline blue to show the callose deposition (lower panel). Bar graph showing the host penetration ability and the percentage of appressoria that formed primary and secondary invasive hyphae from the WT and CPKApromoter*CPK2* at the indicated time points (lower right panel). ^∗^*p* < 0.01, ^∗∗^*p* < 0.001.

Interestingly, the *CPKA*_Promoter_*CPK2* strain produced typical WT-like blast lesions on rice leaves although the lesion size was smaller than the WT lesions (**Figure 7A**), thus indicating that the appressorial function is restored to some extent in the *CPKA*_Promoter_*CPK2* strain unlike in *cpk*AΔ. The *CPKA*_Promoter_*CPK2* appressoria could penetrate the rice leaves, albeit delayed compared to the WT, and were able to successfully invade the host plants. However, the IH growth was compromised and the mutant strain remained restricted to the site of inoculation (**Figure 7B**). Less than 20% of the *CPKA*_Promoter_*CPK2* appressoria could penetrate the rice plants at 28 h (*p* < 0.0001); and by 42 hpi more than 50% appressoria could penetrate (*p* = 0.013) and produce IH with only 10% capable of spread into the neighboring cells. By 42 h, about 80% of the WT appressoria produced secondary IH (*p* = 0.001; **Figure 7B**, bar graph). The *cpkA*Δ was able to produce a successful infection when inoculated through wounds, as observed here and in previous reports. In contrast, the *CPKA*_Promoter_*CPK2* strain penetrated the rice sheath but was defective in IH growth. To check if the suppression of host penetration results from overexpression of *CPK2*, we analyzed the transcript levels of *CPK2* in the *CPKA*_Promoter_*CPK2* strain compared to the WT. The levels of *CPKA* in the *CPKA*_Promoter_*CPK2* strain remained undetectable throughout the vegetative and pathogenic phases thus validating the *cpk*AΔ strain background. Nevertheless, the results confirmed that *CPK2* is not highly expressed in this strain with less than two fold increase at all the phases analyzed, when compared to WT *M. oryzae* (**Supplementary Figure S3**). We infer that the suppression of defects associated with loss of *CPKA* are likely due to the redundancy in Cpk2 function during pathogenic differentiation, and not due to an increase in *CPK2* transcript levels *per se* in the swapped strain. To conclude, Cpk2 shares a potentially redundant function with CpkA, but is unable to fully compensate/complement the loss of *CPKA* activity in *M. oryzae*.

### Cpk2-GFP Localizes to Dynamic Cytoplasmic Vesicles, and the Nucleus

To analyze the subcellular localization of Cpk2 during asexual and pathogenic phases of *M. oryzae*, the Cpk2 was fused with GFP at its C terminus. The expression from the native Cpk2 promoter was too weak to observe proper epifluorescence (**Supplementary Figure S4**). Hence, the GFP-Cpk2 fusion was expressed under the control of the constitutive Histone H3 promoter (*pH3GFP-CPK2*). The *in vivo* functionality of the fusion protein was verified through rigorous analysis of several phenotypes, and the aforementioned modified strains were found to be comparable to the parental untagged strain in all aspects of growth and pathogenicity (**Supplementary Figure S5**). A double-tagged strain, *CPK2-GFP CPKA-mCherry*, was generated to examine the colocalization (if any).

**FIGURE 8:**
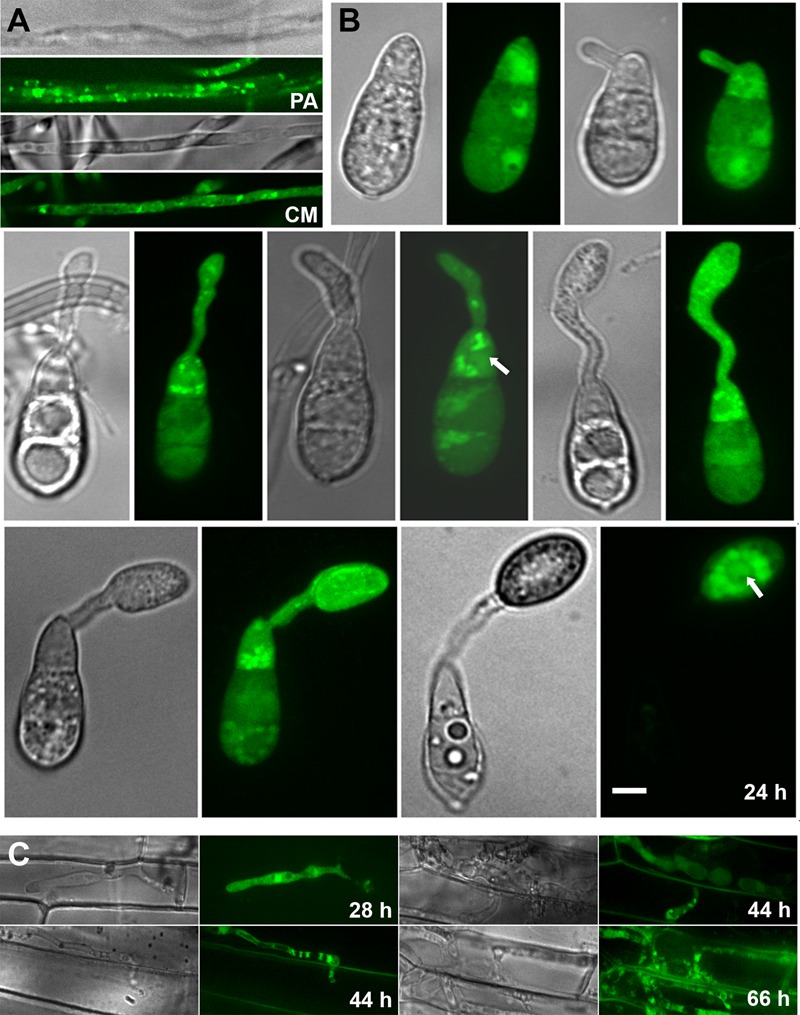
Subcellular localization of the GFP-Cpk2 at different stages of vegetative and asexual development in *M. oryzae*. **(A)** Vegetative hyphae and aerial structures from pH3GFP-CPK2 strain were imaged after growth on PA or CM medium for 3 days. **(B)** Conidia inoculated on the inductive surface were subjected to time lapse analysis and the Bright field and epifluorescent images were captured at the indicated time points using the requisite filters. Images are maximum intensity Z-projections of five confocal stacks, measuring 0.5 μm each. Scale bar is 10 μm. Arrows indicate the nuclear localization of GFP-Cpk2. **(C)** Subcellular localization of GFP-Cpk2 in the invasive hyphae formed in rice leaf sheath at the indicated time points. Scale bar is 10 μm.

**FIGURE 9:**
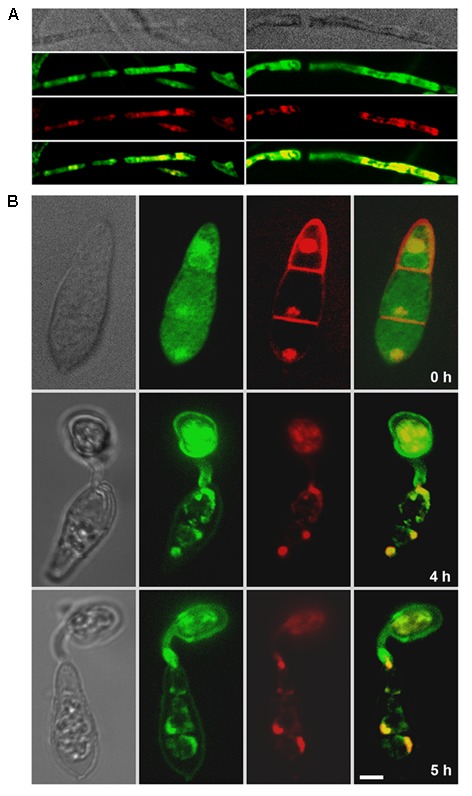
Confirmation of the nuclear localization of GFP-Cpk2. GFP-Cpk2 co-localizes with the nuclear stain DAPI at different structures as observed. Mycelia **(A)** or the conidia **(B)** were inoculated on coverslips and DAPI staining was carried out on fixed samples as described in methods. The co-localization (remains yellow in the merged panel) of GFP-Cpk2 (green) with nuclei/DAPI stained (pseudocolored red) is readily visible at the time points indicated. Images are maximum intensity Z-projections of five confocal stacks, 0.5 μm each. Scale bar equals 10 μm.

**FIGURE 10:**
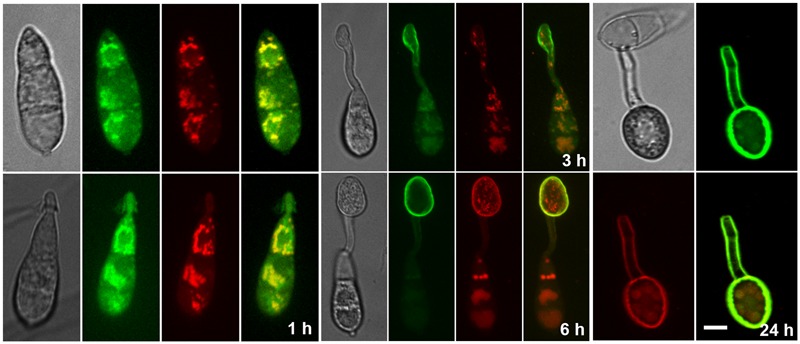
Co-localization of Cpk2-GFP with CPKA-mcherry. Cpk2-GFP cytoplasmic vesicles colocalize with CpkA-mCherry in the conidia and germ tubes. In the appressoria (24 h) a weak co-localization is evident in the vesicular structures within the perinuclear region.

The Cpk2-GFP was highly expressed in the vegetative stage, i.e., in mycelia/aerial hyphae on PA medium, compared to conidia and related structures therein (**Supplementary Figures S4B,C**). Staining with the Hoechst dye confirmed the nuclear localization of Cpk2-GFP during vegetative growth and also in the conidiophores (**Supplementary Figure S4D**). However, such significant colocalization was not evident in the developing conidia due to weak Cpk2GFP signal therein. The constitutively expressed GFP-Cpk2 (*H3Pro*G*FP-CPK2* strain) showed a similar localization pattern, i.e., remained nuclear and cytoplasmic during mycelial growth. Nuclear localization of GFP-Cpk2 was clearly evident in conidia, and as cytoplasmic vesicles in the terminal cell of the conidia and also in germ tubes (**Figure 8**). The GFP-Cpk2 vesicles moved to the emerging appressorium at the hooking stage. In mature appressoria (24 h), GFP-Cpk2 showed a peri-nuclear vesicular localization, although the nuclear localization *per se* was not as prominent as in conidia (**Figure 8**). In order to confirm the nuclear localization of GFP-Cpk2, the conidia from *H3ProGFP-CPK2* strain were co-stained with DAPI. GFP-Cpk2 colocalized with the DAPI signal, thus confirming the nuclear localization of Cpk2 therein (**Figure 9**). The Cpk2-GFP vesicles co-localized with the CpkA-mCherry vesicles in the cytoplasm at different stages analyzed. However, nuclear localization of Cpk2-GFP was not clear in this strain likely because of the weak Cpk2-GFP signal due to native expression, and/or due to masking by the stronger CpkA-mC expression (**Figure 10**). We conclude that Cpk2 is compartmentalized in the nucleus, and its colocalization with CpkA is exclusive to the cytoplasmic vesicles. We infer that such intracellular localization facilitates RpkA interaction with both Cpk2 and CpkA, thus enabling robust cAMP-PKA enzyme activity/function to be regulated effectively in a compartmentalized manner. Lastly, the localization pattern clearly supports some special targets (and/or functions) for Cpk2 in activating the downstream cyclic AMP signaling in the nucleus during pathogenic differentiation in the rice blast fungus.

## Discussion

Most filamentous fungi examined to date, contain multiple isoforms of the catalytic cAMP-PKA subunit, with one such isoform playing a predominant role in growth and development. Despite its importance in regulating biological features and pathogenicity, the PKA catalytic subunits are not essential for cell viability in a number of fungal species (Dürrenberger et al., 1998; D’Souza and Heitman, 2001; Fuller et al., 2011). However, at least one TPK gene is required for cell viability and the *tpk1 tpk2 tpk3* triple mutant is not viable in *S. cerevisiae* (Toda et al., 1987). In *A. nidulans*, deletion of both *pkaB* and *pkaA* is lethal, though overexpression of *pkaB* can suppress some defects caused by Δ*pkaA*, indicating the overlapping roles of PkaA and PkaB (Ni et al., 2005). *U. maydis* and *F. graminearum* represent only a minority of plant pathogens wherein the effect of the loss of both the PKA-C isoforms has been analyzed (Dürrenberger et al., 1998; Hu et al., 2014). *M. oryzae* contains a divergent PKA-C isoform, Cpk2, but its contribution to growth and pathogenesis remained unexplored largely due to the earlier indication that the *cpkAcpk2* double mutant is likely inviable (Choi and Xu, 2010). A major challenge in deciphering the overall cAMP-PKA signaling is to fully understand the contribution(s) of both the catalytic subunits, CpkA and Cpk2, in *M. oryzae*. Previous studies revealed the importance of CPKA in appressorium formation and pathogenicity in *M. oryzae* (Mitchell and Dean, 1995; Xu et al., 1997; Selvaraj et al., 2017). Our study provides a detailed elucidation of the function, dynamics and organization of the individual signaling components, CpkA and Cpk2 of the cAMP-PKA signaling in *M. oryzae*. Here, we showed that the cAMP-PKA signaling is not required for viability *per se* in *M. oryzae*, but is necessary for proper growth, conidiation and pathogenicity. This further adds to the previous findings that *MAC1* or *RPKA* are dispensable for viability but essential for pathogenic differentiation (Choi and Dean, 1997; Selvaraj et al., 2017). Our study also complements a recent elegant analysis (Li Y. et al., 2017) of an important downstream transcriptional target/effector of PKA-C, *MoSFL1*, which was identified as a spontaneous suppressor in the double deletion mutant of the cAMP-PKA catalytic subunits. In addition, we have provided a detailed functional analysis and subcellular localization of Cpk2, and gained insight into the functional interdependency between the two PKA-C subunits, which had remained unelucidated thus far in *M. oryzae*.

In *M. oryzae*, CpkA plays a role in appressorium morphogenesis and plant infection though it is dispensable for vegetative growth and conidiation, and is predicted to contribute the major PKA catalytic function (Mitchell and Dean, 1995; Xu et al., 1997). Consistent with this observation, deletion of *CPK2* had no detectable effect on conidiation, appressorium formation or pathogenicity, thus making it redundant in function with *CPKA*. The enzyme activities and relative expression of the two isoforms in WT and mutant backgrounds revealed that cells do not compensate for the loss of one C isoform by overexpression of the other as in *S. cerevisiae* (Mazón et al., 1993; Robertson et al., 2000) and their interactions are certainly not regulated at the transcriptional level. However, we have shown here the functional capacity of Cpk2 to act in concert with CpkA in the regulation of vegetative growth and conidiation in *M. oryzae.* Appressorium induction upon proper surface sensing is a crucial step for pathogenesis in *M. oryzae*, and our results reflect the importance of Cpk2 function in this process. Our findings suggest that Cpk2, in addition to acting as an inducer of appressorium formation in concert with CpkA, also plays a regulatory role in suppressing appressoria biogenesis under unfavorable conditions such as on hydrophilic surfaces.

The defects of PKA-C null mutant (*cpkA*Δ*cpk2*Δ) resembled the phenotype of *mac1*Δ (Choi and Dean, 1997), despite the significantly higher accumulation of cAMP in this strain and also in individual mutants of the PKA-C subunits. In *S. cerevisiae* and *Cryptococcus neoformans*, it has been shown that the PKA-C regulate cAMP levels through a negative feedback loop by activating PDEs (Ma et al., 1999). Preliminary results on cAMP levels in PKA mutants showed that such feedback inhibition on intracellular cAMP levels via PdeH occurs in *M. oryzae* too. Further, the downregulation of Pmk1 phosphorylation in the PKA-C mutants implies that the crosstalk between the cAMP and MAPK signaling might occur at the level of PKA-C. Although not characterized fully in this study, the defects in *cpkA*Δ*cpk2*Δ related to surface sensing and response, appear similar to the suppressor mutant phenotypes in the *CHM1*-deletion mutant (Li et al., 2004). The molecular identity of such suppressor(s) of *chm1*Δ has not been ascertained yet. The *chm*1Δ also showed additional defects in hyphal growth, conidiation and appressorium formation, which could not be suppressed by exogenous cAMP (Li et al., 2004).

Interestingly, we did not detect phosphorylation of the synthetic PKA substrate, kemptide, in any of the strains in which *CPKA* was deleted. Thus, Cpk2 is unable to serve as the primary PKA-C in *M. oryzae*. The Cpk2 overlap provides a basal level of PKA-C function to allow efficient vegetative growth and to maintain turgor for penetration of appressoria, but not inducible PKA function sufficient for conidiation and appressorium morphogenesis or proper IH growth. Nevertheless, the localization of Cpk2-GFP clearly implies some special functions for *CPK2* in *M. oryzae*. It is well recognized that compartmentalization of cAMP signaling allows spatially distinct pools of PKA to be differentially activated. PKA isoforms are anchored at specific intracellular sites by A-kinase anchoring proteins (AKAPs) in mammalian cells (Smith and Scott, 2006). However, AKAPs are not present in fungi. We showed earlier that cAMP signaling is compartmentalized in the nucleus and cytoplasm in *M. oryzae* (Ramanujam and Naqvi, 2010). The RpkA localizes to the nucleus whereas CpkA is present predominantly on cytoplasmic vesicles with the PKA holoenzyme being cytosolic (Selvaraj et al., 2017). We infer that the nuclear pool of Cpk2-GFP is a consequence of its association with RpkA therein, and that this interaction drives the nuclear function of cAMP signaling in *M. oryzae*. The primary locale for CpkA being vesicular structures in the cytoplasm; and the RpkA and Cpk2 being in the cytoplasm and nucleus allows discrete cAMP-PKA modules that respond to distinct intracellular cAMP pools and subsequently modify specific target proteins. Furthermore, the compartmentalization of cAMP PDEs, the PdeH and PdeL to the cytoplasm and nucleus respectively (Ramanujam and Naqvi, 2010) also shows the importance of tailoring individual cAMP responses to precisely modulate the downstream signaling cascade.

## Conclusion

Proper PKA-C signaling is essential for the invasive growth and pathogenicity, and the balanced activities of the CpkA and Cpk2 isoforms likely plays important roles in robust regulation of the infection process in *M. oryzae*. CpkA being the primary PKA, Cpk2 maintains important function(s) in regulating vegetative growth, conidiation and appressorium formation and also contributes to the spatial and temporal regulation of cAMP-PKA signaling in *M. oryzae*. CpkA and Cpk2 act in a redundant as well as parallel/specific manner to activate the downstream effectors of the cyclic AMP signaling and also Pmk1 MAPK during initiation and spread of the blast disease in rice. Future studies will focus on analyzing the differential regulation and downstream targets of the two PKA-C isoforms in the rice blast pathosystem.

## Author Contributions

Conceived and designed the experiments: NN. Performed the experiments: PS, QS, and FY. Analyzed the data: PS and NN. Contributed reagents/materials/analysis tools: NN. Wrote the paper: PS and NN.

## Conflict of Interest Statement

The authors declare that the research was conducted in the absence of any commercial or financial relationships that could be construed as a potential conflict of interest.

## Footnotes

1 http://www.ch.embnet.org/software/BOX_form.html

2 http://fiji.sc/wiki/index.php/Fiji

3 http://fungi.ensembl.org/Magnaporthe_oryzae/Gene/Summary

## Supplementary Material

FIGURE S1 Amino acid sequence alignment for the PKA-C subunits, CpkA and Cpk2, from *M. oryzae*. The protein sequences of CpkA and Cpk2 showing identities (shaded black) and similarities (gray background). Alignment was carried out using ClustalW of the MegAlign software, a program of the Lasergene package (DNASTAR) using default parameters; and shaded with BOXSHADE 3.2.1.

FIGURE S2 Construction of deletion mutants in *CPKA* and *CPK2* mutants and confirmation of the transformants. **(A)** Schematic representation of *CPKA* (left) and *CPK2* (right) loci, and the deletion mutant strategy *in M. oryzae*. The open reading frame of the genes was deleted and replaced with the *Glufosinate ammonium* (BAR) or *hygromycin resistance* (HPH) cassette. Solid bars and short open boxes represent coding regions and introns, respectively; whereas dashed lines indicate the genomic flanks used for gene targeting. Restriction endonuclease cleavage sites (E- EcoRI, B- BamHI, P-PstI, H-HindIII). **(B)** Southern blot analysis of the genomic DNA of WT and transformants for CPKA (1–5) (left) and CPK2 (1–7) (right) transformants. **(C)** Locus specific PCR confirming the proper integration of *cpk2* deletion in *cpk*AΔ background.

FIGURE S3 The *CPK2* is not overexpressed in WT when driven with the *CPKA* promoter. Bar graph showing the fold change in the transcript levels of *CPKA* and *CPK2* in the *CPKApromoterCPK2* strain at different time points analyzed during vegetative or pathogenic growth. Fold change in gene expression was calculated from the average of three independent measurements, with β-Tubulin of *M. oryzae* as internal control and normalized to WT *CPKA* and *CPK2* levels at the respective time points.

FIGURE S4 The Cpk2-GFP is weakly expressed with the native promoter. **(A)** Schematic representation showing the in-frame integration of *GFP* within the *CPK2* genomic locus; and the locus-specific PCR of the transformants confirming the requisite integration. **(B)** Confocal images showing the cytoplasmic localization of Cpk2-GFP in the hyphae grown in Prune agar or Complete medium. **(C)** Cytoplasmic and nuclear localization of Cpk2-GFP in conidia at different stages of pathogenic differentiation. Arrows indicate nuclear localization of the Cpk2-GFP. **(D)** Images showing colocalization of Cpk2-GFP (green) with the Hoechst stain (nuclear marker - pseudocolored in blue) in the mycelia and conidiophore.

FIGURE S5 Characterization of *GFP-CPK*2 strain. The strain *H3ProGFP-CPK2* forms appressoria on inductive and non-inductive surfaces **(A)** and able to penetrate the rice leaf sheath and produce the infection similar to the WT *M. oryzae* **(B)**.
