# Supplementary material for: Cpk2, a Catalytic Subunit of Cyclic AMP-PKA, Regulates Growth and Pathogenesis in Rice Blast
Source: Front Microbiol. 2017 Nov 21;8:2289. doi: 10.3389/fmicb.2017.02289 (PMC5702331; doi:10.3389/fmicb.2017.02289)
Supplement: FIGURE S1 — Amino acid sequence alignment for the PKA-C subunits, CpkA and Cpk2, from M. oryzae. The protein sequences of CpkA and Cpk2 showing identities (shaded black) and similarities (gray background). Alignment was carried out using ClustalW of the MegAlign software, a program of the Lasergene package (DNASTAR) using default parameters; and shaded with BOXSHADE 3.2.1. [file Image_1.PDF]

```
cpk2    1 ------------------------------------------------------------
cpkA    1 MPSLGFLKKKRTRDGNNDNSSQPASPVTPTAAQSFEQAQVLGAPSAINNSHAHTQQQSYL

cpk2    1 ------------------------------------------------------MAAALA
cpkA   61 VPQPGYSVGTEVQAQQPQMNSISQQQQQAFAPPAHTPSPGTIDPQQSLPSISNLMNPAVQ
                                                                *  *.

cpk2    7 H--------------SSGMIPSMVGVPPSTSYENGSGGNSSTT----------------A
cpkA  121 QQNSQPSANFQPQSQSQSQSQSQFPLPPSHG----NGDQSQQQNFQVQQQIQSQQDAMDI
                         *     *   .***  .... * .*

cpk2   37 GHGSVNDGSR--------TSRNALQGDL-----KSDAASAQSQNRKLQLSDFKKVRTLGT
cpkA  177 QPSQVQDQSHSQQAQPQHQPQHHVQHHVNHAHQGSQDQQTRVTKGKYSLTDFEILRTLGT
              *.* *.........     .*  ......  *          *  *.**  .*****

cpk2   84 GTFARVCLVRPSNPQNETERNKVFALKILRKSEVVKLKQIDHVRHERAILADVSGFPFIT
cpkA  237 GSFGRVHLVQSRH------NQRFYAVKVLKKAQVVKMKQVEHTNDERKMLGEVK-NPFLI
          *.*.** **    ...... .. .*.*.*.*  ***.**..*   ** .*..*  .**.

cpk2  144 NMLASFSDHDFLYIVLDYVPGGELFSYLRKYRRFDEDMARFYAAEIVLVLEYLHEAQDGV
cpkA  290 TLWGTFQDCRNLYMVMDFVEGGELFSLLRKSGRFPNPVAKFYAAEVTLALEYLHA--KNI
           . ..* *   **.*.*.* ****** ***  **   .*.*****. * ***** ..  .

cpk2  204 AYRDLKPENLLLDGQGHIKLVDFGFAKRLGGRRDGDNSGTQETYTLCGTPEYLAPEVIHN
cpkA  348 IYRDLKPENLLLDRHGHLKITDFGFAKRVP----------DKTWTLCGTPDYLAPEVVSN
           ************  **.*. *******.  ..........  *.******.******. *

cpk2  264 KGHTTAVDWWALGILIYEFLTGYPPFWHQ-NPIEIYKQIVEKPVVFPQDPPISPNAQDII
cpkA  398 KGYNKSVDWWSLGILIYEMLCGYTPFWDSGSPMKIYENILKGKVRYPA--YINPDAQDLL
          **    **** ******* * ** ***  . *. ** .*.   * .*.. * * ***..

cpk2  323 RQFCTVDRSRRLGNISGGAARVKEHPFFEGVDWEAIYSRKFPGPILPPIRY-PGDAQCFD
cpkA  456 QRLITADLTKRLGNLYGGSQDVRNHPWFAEVTWDRLARKDIDAPYTPPVKAGAGDASQFD
              * * ..****. **   *. **.*  * *. .  .   .*  **.. . ***  **

cpk2  382 IYPEEDVGKDPYTDEMAQKYDHYFQDF
cpkA  516 RYPEETER---YGQTGHDEYGNLFPGF
           ****   ...*        *   *  *
```

Fig. S1

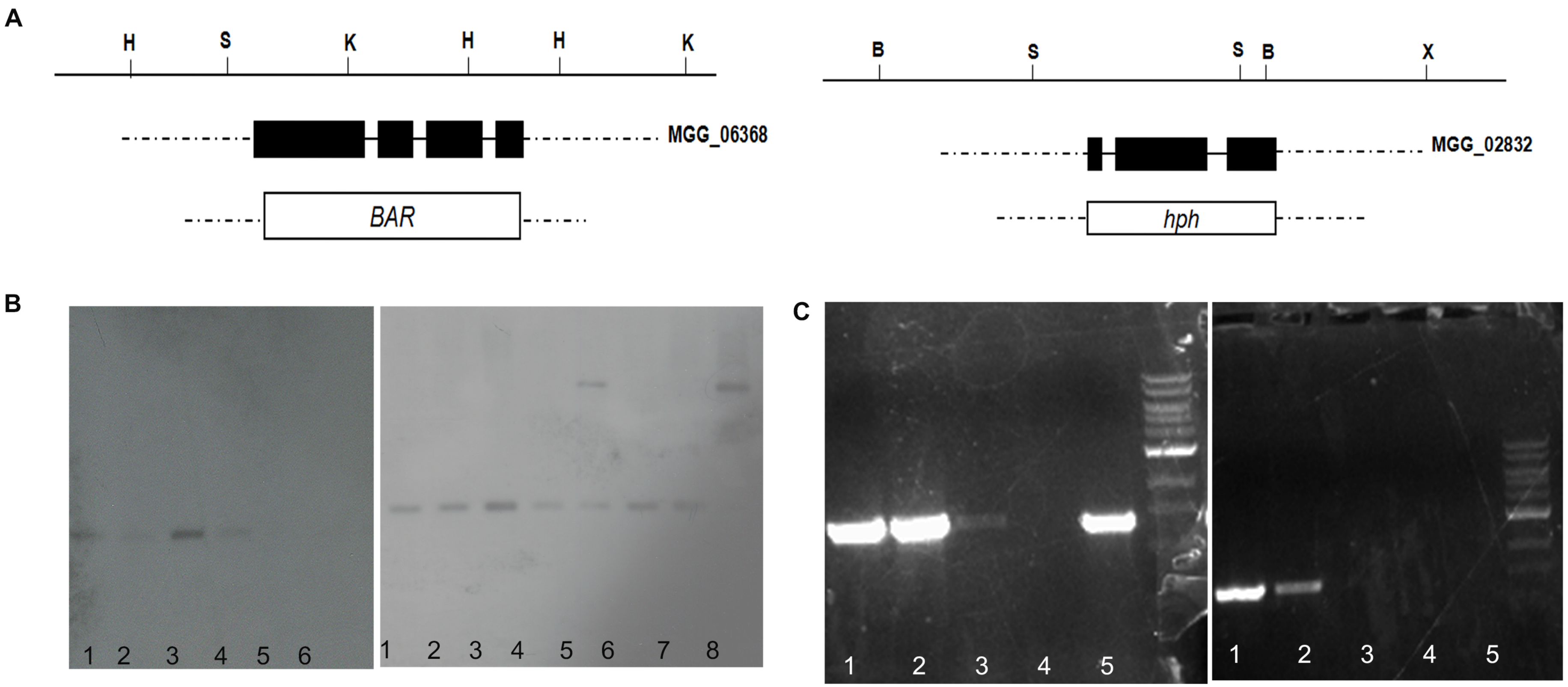
A
H
S
K
H
H
K
MGG_06368
BAR
B
S
S
B
X
MGG_02832
hph
B
1 2 3 4 5 6
1 2 3 4 5 6 7 8
C
1 2 3 4 5
1 2 3 4 5

Fig. S2

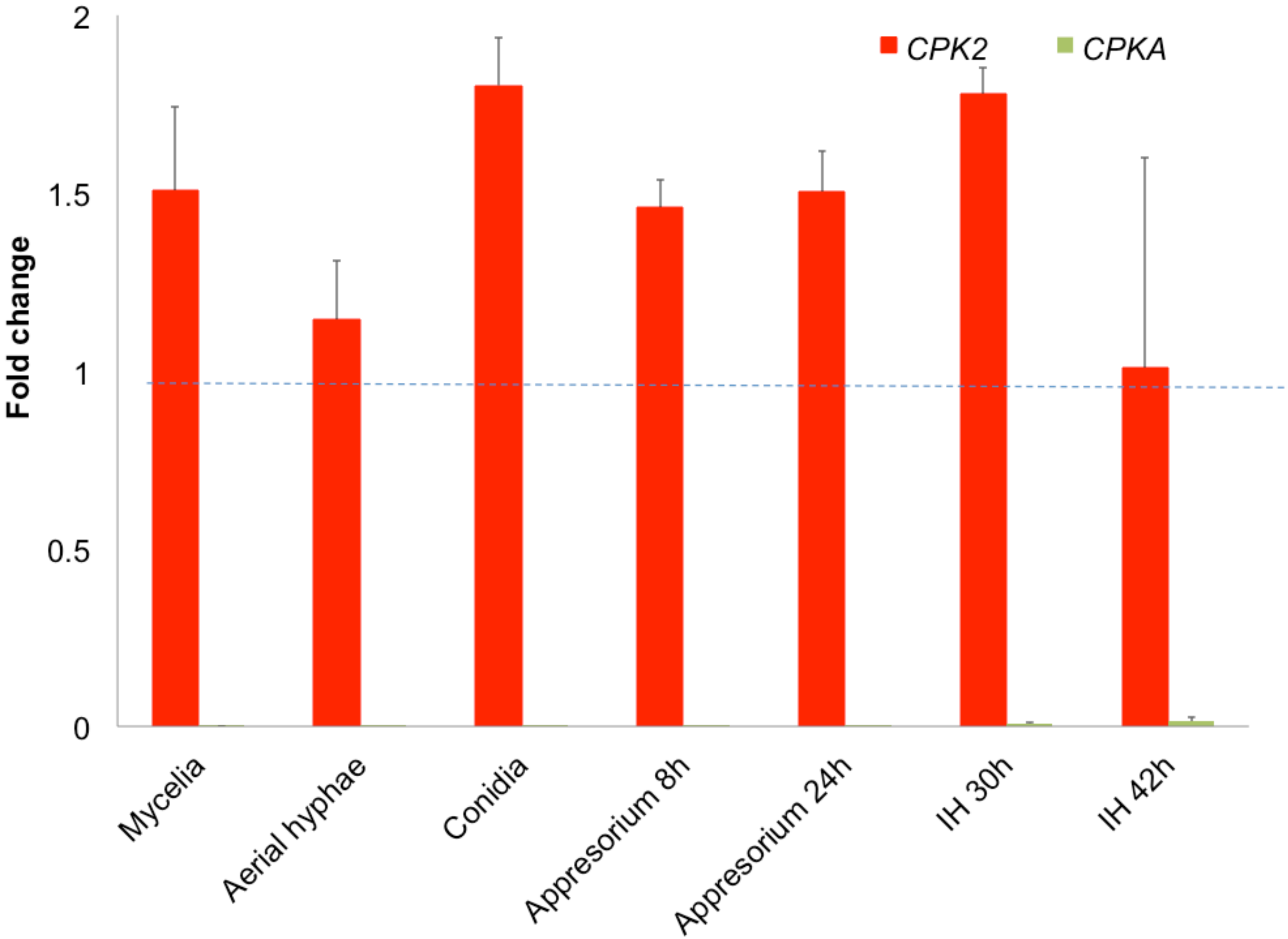

2
1.5
1
0.5
0
Fold change
CPK2
CPKA
Mycelia
Aerial hyphae
Conidia
Appresorium 8h
Appresorium 24h
IH 30h
IH 42h

Fig. S3

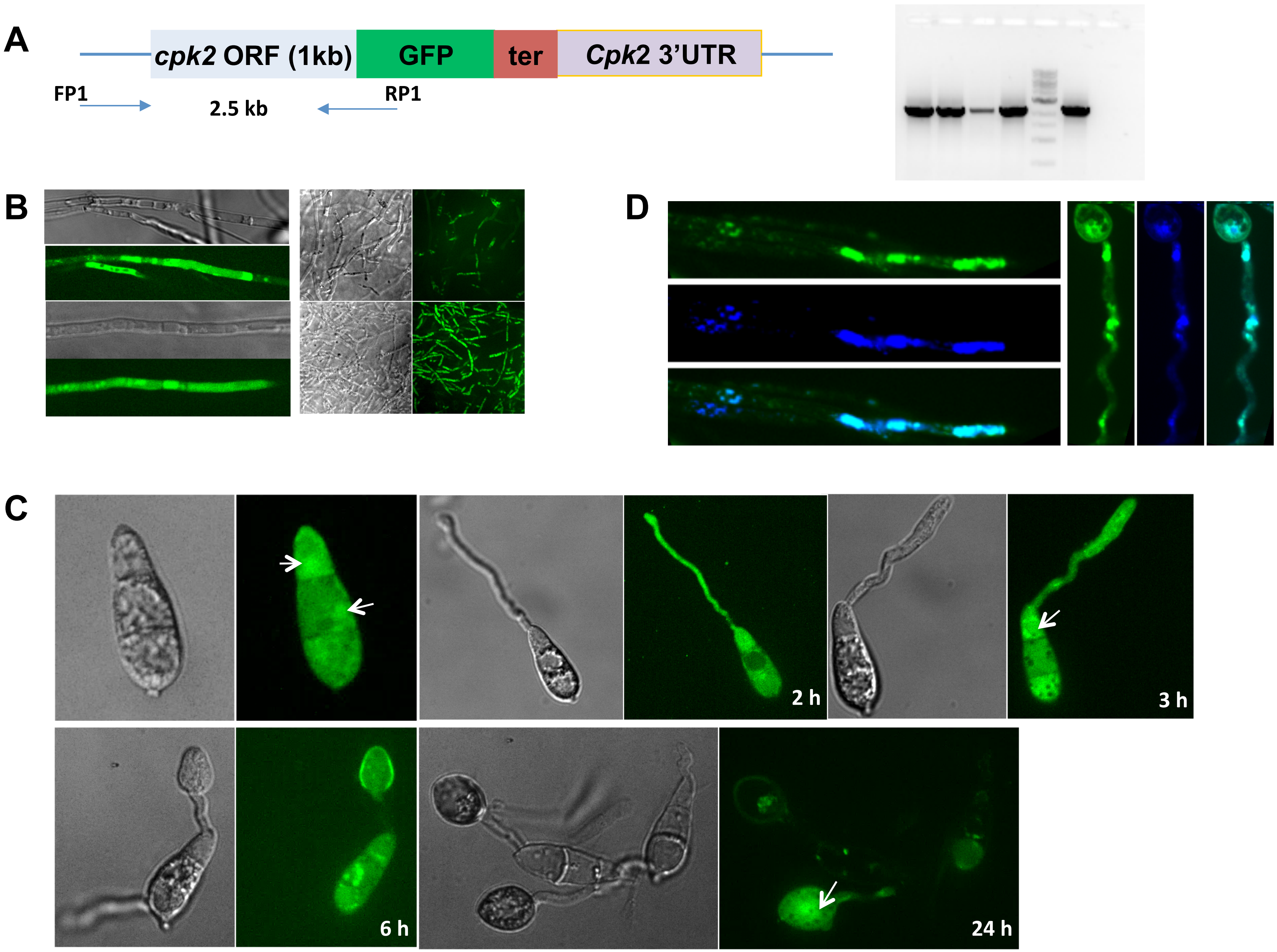

Fig. S4

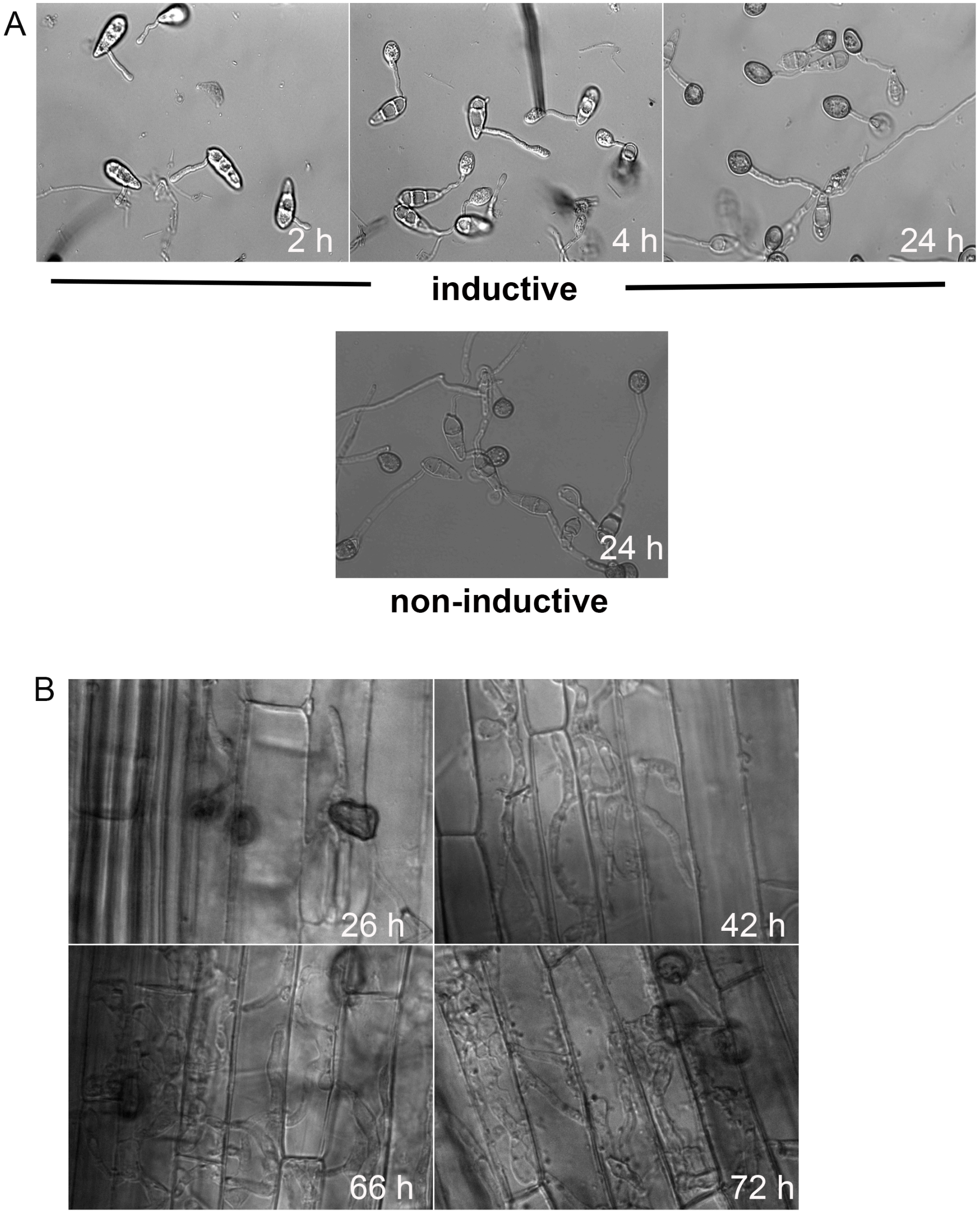

Fig. S5
