# Supplementary material for: Cpk2, a Catalytic Subunit of Cyclic AMP-PKA, Regulates Growth and Pathogenesis in Rice Blast
Source: Front Microbiol. 2017 Nov 21;8:2289. doi: 10.3389/fmicb.2017.02289 (PMC5702331; doi:10.3389/fmicb.2017.02289)
Supplement: Supplementary file 2 [file Table_1.DOCX]

**Table S1. List of oligonucleotide primers. Restriction enzyme sites introduced for cloning purposes are underlined.**

| **Description** | **Gene** | **Enzyme site** | **Oligonucleotide sequence of Primer (5’-3’)** |
| --- | --- | --- | --- |
| Deletion construct for *CPK*2 | *CPK2* 5′ UTR | EcoRI | CAGAGAGAATTCTGGCTTGGATCTGCCCTC (F) |
|  |  | BamHI | CAGAGAGGATCCGGAAGGAATCATCCCCGA (R) |
|  | *CPK2* 3′ UTR | PstI | CAGAGACTGCAGCTGTCTCATTGTGGCGCTTG (F) |
|  |  | HindIII | CAGAGAAAGCTTCTGGCATATGGCATGTGGTA (R) |
| *Locus specific PCR for* CPK2 deletion | *CPK2* 5′ UTR | FP | AACTCAATCCCGCCTTGTCC |
|  | *CPK2* 3′ UTR | RP | TTCCTCCTCTAGAGCACTTG |
| *CPK*2 C-terminal tagging with GFP | *CPK2* ORF 1kb | EcoR1 | CAGAGAGAATTCATGGCGGCTGCTCTG (F) |
|  |  | EcoRI | CAGAGAGAATTCAAAGTCCTGAAAGTAGTGGTCG (R) |
|  | GFP | EcoRI | CAGAGACCATGGTGAGCAAGGGCGAGGAGCTGT (F) |
|  |  | BamHI | CAGAGACCCGGGTTACTTGTACAGCTCGTCCATGCC (R) |
|  | TrpC terminator | BamHI | CAGAGAGGATCCACTTAACGTTACTGAAATCATCAA(F) |
|  |  | XbaI | CAGAGATCTAGACGAGCCCTCTAAACAAGTGT(R) |
|  | gfp | RP | TGAAGGGCATCGACTTCAAGG |
|  | CPk2 DS | RP |  |
| *CPK*2 tagging with GFP with Histone 3 promoter | Promoter H3 | EcoRI | TGTGAATTCGTGGGGGACGACCTTACCT |
|  |  | BamHI | GGGGGATCCTGATTGATTTGTGATTGATGAAAA |
|  | *CPK2* ORF | XbaI | CAGAGATCTAGA ATGGCGGCTGCTCTG |
|  |  | PStI | CAGAGACTGCAGAAAGTCCTGAAAGTAGTGGTCG |
|  | *GFP* | BamHI | CAGAGAGGATCCATGGTGAGCAAGGGCGAGGA |
|  |  | XbaI | CAGAGATCTAGACTTGTACAGCTCGTCCATGCC |
| Cpk2 driven by cpkA promoter for complementing CPKA | *CPK*A 5′ UTR | EcoRI | CAGAGAGAATTCTCGGCTTGCTTATCCCTT (F) |
|  |  | SpeI | CAGAGAACTAGTCGAGGCGACAATGGGGATT (R) |
|  | *CPK*A 3′ UTR | PstI | CAGAGACTGCAGAGAGGCTATGATTTGTATTCCAC (F) |
|  |  | HindIII | CAGAGAAAGCTTCAT ACCTACTGTATTCAGT (R) |
|  | *CPK*2 ORF | SpeI | CAGAGAACTAGTATGGCGGCTGCTCTG |
|  |  | XbaI | CAGAGATCTAGAAAAGTCCTGAAAGTAGTGGTCG |
| *Real time qPCR* | | | |
| CPKA | TGCTGTGCGGCTATACCCCCTTCT (F)  CGCGCCGGCCTTGACTGGAG (R) | | |
| CPK2 | GGAACCCAGGAGACGTACAC (F)  GGCTTTTCGACGATCTGCTTG (R) | | |
| β - Tubulin | ACTGATGTCGCTGTTCTTCT (F)  CTGTTGAGGTTGGTGTAGGT (R) | | |
